# Digital Public Health Solutions in Response to the COVID-19 Pandemic: Comparative Analysis of Contact Tracing Solutions Deployed in Japan and Germany

**DOI:** 10.2196/44966

**Published:** 2023-06-14

**Authors:** Candice Louw

**Affiliations:** 1 Department of Epidemiology Helmholtz Centre for Infection Research Braunschweig Germany

**Keywords:** contact tracing, COVID-19, digital health, digitalization, open-source software, pandemic preparedness, pandemic technologies

## Abstract

**Background:**

In response to the COVID-19 pandemic, numerous countries, including the likes of Japan and Germany, initiated, developed, and deployed digital contact tracing solutions in an effort to detect and interrupt COVID-19 transmission chains. These initiatives indicated the willingness of both the Japanese and German governments to support eHealth solution development for public health; however, end user acceptance, trust, and willingness to make use of the solutions delivered through these initiatives are critical to their success. Through a case-based analysis of contact tracing solutions deployed in Japan and Germany during the COVID-19 pandemic we may gain valuable perspectives on the transnational role of digital technologies in crises, while also projecting possible directions for future pandemic technologies.

**Objective:**

In this study, we investigate (1) which types of digital contact tracing solutions were developed and deployed by the Japanese and German governments in response to the COVID-19 pandemic and (2) how many of these solutions are open-source software (OSS) solutions. Our objective is to establish not only the type of applications that may be needed in response to a pandemic from the perspective of 2 geographically diverse, world-leading economies but also how prevalent OSS pandemic technology development has been in this context.

**Methods:**

We analyze the official government websites of Japan and Germany to identify digital solutions that are developed and deployed for contact tracing purposes (for any length of time) during the timeframe January-December 2021, specifically in response to the COVID-19 pandemic. We subsequently perform a case-oriented comparative analysis, also identifying which solutions are published as open-source.

**Results:**

In Japan, a proximity tracing tool (COVID-19 Contact-Confirming Application [COCOA]) and an outbreak management tool (Health Center Real-time Information-sharing System on COVID-19 [HER-SYS]) with an integrated symptom tracking tool (My HER-SYS) were developed. In Germany, a proximity tracing tool (Corona-Warn-App) and an outbreak management tool (Surveillance Outbreak Response Management and Analysis System [SORMAS]) were developed. From these identified solutions, COCOA, Corona-Warn-App, and SORMAS were published as open-source, indicating support by both the Japanese and German governments for OSS pandemic technology development in the context of public health.

**Conclusions:**

Japan and Germany showed support for developing and deploying not only digital contact tracing solutions but also OSS digital contact tracing solutions in response to the COVID-19 pandemic. Despite the open nature of such OSS solutions’ source code, software solutions (both OSS and non-OSS) are only as transparent as the live or production environment where their processed data is hosted or stored. Software development and live software hosting are thus 2 sides of the same coin. It is nonetheless arguable that OSS pandemic technology solutions for public health are a step in the right direction for enhanced transparency in the interest of the greater public good.

## Introduction

In response to the COVID-19 pandemic, numerous countries, including the likes of Japan and Germany, initiated, developed, and deployed digital contact tracing solutions in an effort to detect and interrupt COVID-19 transmission chains. During such contact tracing activities, individuals who were recently in contact with an infected person are identified in order to place them in quarantine or isolate them in an effort to reduce further disease transmission [1]. Incremental investment in contact tracing may be highly advantageous, as it may yield diminishing reductions in disease prevalence [2]. This is due to contact tracing initiatives being the first and often considered to be the most effective step toward epidemic control, as resources for mass testing and large amounts of vaccines may still be unavailable at the time of the initial outbreak [3]. Moreover, contact tracing, followed by quarantine, isolation, or treatment, are key control measures in the battle against infectious diseases and may be particularly efficient when dealing with low numbers of cases [4]. As the use of digital technology may enhance and accelerate manual contact tracing efforts [1], adopting or implementing digital contact tracing solutions may become of particular interest during outbreaks that result in high volumes of cases and contacts, such as during the COVID-19 pandemic.

Digital contact tracing solutions that may offer support in this regard are categorized into 3 main categories by the World Health Organization, namely, (1) outbreak response and data collection and management tools, (2) proximity tracing tools, and (3) symptom tracking tools [1,5]. While outbreak response and data collection and management tools require active capturing and management of case and contact data in an electronic system, proximity tracing tools focus on tracing the movements of individuals to retrospectively notify them if they had been in contact with an infected person [1]. Symptom tracking tools, in turn, allow the capture of self-reported signs and symptoms by end users in an attempt to inform the contact tracing process [1,5]. These activities may be performed by local health authorities that actively gather the data or by end users (typically the public) who actively share their data with the local health authorities or others (public-pushed data).

Regardless of the perspective from which health data are pulled or pushed, the support for implementing and encouraging the adoption of such digital contact tracing solutions during the COVID-19 pandemic in Japan and Germany indicated the willingness of both the Japanese and German governments to support eHealth solution development for public health. While these 2 high-tech countries were (in 2022) the world’s third (Japan) and fourth (Germany) largest economies, respectively, they are roughly also the same size at 377,975 km^2^ (Japan) and 357,582 km^2^ (Germany) [6]. Despite the differences in land shape, geographic location, system of government (Japan: unitary; Germany: federal), and the fact that Japan has more inhabitants (126 million) than Germany (83 million), both countries are parliamentary democracies and free market economies that believe in multilateralism and global trade [6]. As a result, identifying and analyzing the digital contact tracing tools developed by these 2 countries in response to the COVID-19 pandemic may provide perspectives on the transnational role of digital technologies in crises while also providing preliminary results to potentially inform strategies for future pandemic technologies from the perspective of 2 geographically diverse, world-leading economies.

End user acceptance, trust, and willingness to make use of the digital solutions delivered through such government initiatives are, however, critical to not only their immediate but also sustained future success. Trust in government has strongly been correlated with end user willingness to use a specific contact tracing application, and as such, government communication during a public health crisis should focus on building or restoring trust among the population to encourage them to accept and support the usage of such applications [7].

In an attempt to further such trust with open-source software (OSS) solutions, contrary to non-OSS or closed-source software (CSS) and proprietary software solutions, an opportunity exists to be transparent during the software’s development life cycle (at least to more of an extent than with CSS). This is possible with OSS solutions as the source code (the instructions that programmers write that dictate how a piece of software behaves) is published on a public platform for anyone to scrutinize at any time. Moreover, OSS licenses make OSS more affordable compared to CSS, thereby making open-source licensing for public health systems essential to a rational procurement strategy [8]. Favorable initial feature response times, development scale-up and scale-down possibilities, prevention of vendor lock-in situations, and the possibility to drive down costs speak to further advantages of implementing OSS solutions for public health [8,9].

Public health is, however, political by nature and needs to be political in order to influence national policy [10]. This does mean that public health is vulnerable to criticism, which could potentially result in reduced funding when political decisions go wrong [10]. Fortunately, with OSS solutions for public health, past development efforts are not lost in the event of funding being lost. Instead, past development efforts remain available to the global community to benefit from while also laying the foundation for future development by other OSS solution contributors, independent of a single source of funding. As such, OSS is recognized as a social and economic phenomenon that raises fundamental questions about the motivations of contributors [11] with social-relational factors having been found to be very important to promote a developer's participation in an OSS project [12].

In order to determine whether a particular software solution is truly open source, the following 3 guiding questions may be asked: (1) Is the source code hosted on a public repository? (2) Is the source code visible & accessible to the public for input, feedback, and interaction? (3) Is a high level of independent source code customization possible as dictated by an appropriate OSS license?

In this study, we subsequently investigate (1) which types of digital contact tracing solutions were developed and deployed by the Japanese and German governments in response to the COVID-19 pandemic and (2) how many of these solutions are open source. By answering these research questions, we gain insight into not only the type of solutions that have been developed in response to a pandemic from the perspective of 2 geographically diverse, world-leading economies but also how prevalent OSS pandemic technology development has been in this context.

We commence our investigation by identifying the digital contact tracing solutions deployed in response to the COVID-19 pandemic in Japan and Germany by the respective governments for use by local health authorities and end users in the respective countries. Furthermore, we use the aforementioned guiding questions to identify which of the selected solutions are OSS, subsequently also analyzing the software publication and management approaches taken by the identified OSS initiatives. We delve into the chosen methods to accomplish this next.

## Methods

In this study, we follow a qualitative, case-based, comparative research method in which we identify, select, classify, and analyze a small sample of cases [13]. A case-oriented comparative analysis, which entails the observation of several different variables across the aforementioned cases [14], subsequently follows.

We commence the process by analyzing the official government websites of Japan (Ministry of Health, Labor, and Welfare) [15] and Germany (Bundesministerium für Gesundheit) [16] and identifying digital solutions that have been implemented and used for contact tracing purposes (for any length of time) during the timeframe January-December 2021, specifically in response to the COVID-19 pandemic. Based on these selection criteria, the solutions included in the case sample are subsequently the COVID-19 Contact-Confirming Application (COCOA) deployed in Japan [17], the Health Center Real-time Information-sharing System on COVID-19 (HER-SYS, also including MyHER-SYS) deployed in Japan [18,19], the Corona-Warn-App deployed in Germany [20], and the Surveillance Outbreak Response Management and Analysis System (SORMAS) deployed in Germany [21]. Next, each of the identified solutions is classified (as discussed in the introduction) as an outbreak response and data collection and management tool, proximity tracing tool, or symptom tracking tool, also indicating if it is primarily used by health authorities or the public. During this analysis, we indicate whether a solution is open source by making use of the guiding questions for identifying OSS solutions as summarized in Table 1 (also previously discussed in the introduction).

Finally, through a comparative analysis (primarily by method of agreement) [22], we identify the similarities between the open-source solutions’ source code publication and management approaches. We analyze each of the identified solutions in more detail and elaborate on the obtained results next.

**Table 1:** Open-source software (OSS) identifying characteristics and guiding questions.

Identifying question	OSS	CSS^a^
1. Source code published on a publicly accessible repository?	Yes	No
2. Source code accessible for public input, feedback, or interaction?	Yes/No	No
3. High level of independent or public source code customization possible as dictated by appropriate OSS license?	Yes	No

^a^CSS: closed-source software.

## Results

COCOA is a smartphone app for Android and iOS devices that requires end users to voluntarily install the app on their own devices. By means of Bluetooth technology, the app automatically records close contacts with other people also using the app within a distance of 1 meter for at least 15 minutes [23]. Based on this behavior, the app can be classified as a proximity tracing app, with end users voluntarily installing the app on their smartphones and voluntarily pushing their data through the app. Upon a closer inspection of the source code, it becomes clear that it is hosted on a public repository on GitHub and contains information about the repository in both English and Japanese. The source code is available to the public to view, and public interaction with the repository owners (developers) of the source code is possible with instructions provided on the repository in the form of a ReadMe file. Public users are able to create their own version of the software or contribute to the existing software source code by following the instructions provided in the same ReadMe file. Finally, the software is published under the open-source license Mozilla Public License version 2.0.

HER-SYS is a web-based public health surveillance tool that allows information on COVID-19–infected patients (symptoms and travel history) to be input digitally, controlled centrally, and shared among the relevant personnel (including doctors, health authorities, and local governments) [24,25]. It is typically installed and used by local health authorities and can therefore be classified as an outbreak management tool, although the MyHER-SYS web-based application provides an extension point to the HER-SYS system that allows patients to report their own data (symptoms) to the HER-SYS system (if previously registered). This approach allows the digitalization and realization of the once-only principle for on-site work by local public health center personnel [24] and introduces the additional classification as a symptom tracking tool. The software source code is not available on the internet for either HER-SYS or MyHER-SYS, which implies that they are proprietary or CSS.

The Corona-Warn-App is similar to COCOA and is a smartphone app for Android and iOS devices that requires end users to voluntarily install the app on their own devices. By means of Bluetooth Low Energy Technology, other app users in proximity are detected by exchanging encrypted identification numbers between devices, thereby informing users whether they have been in contact with an infected person who also makes use of the app [26]. Based on this behavior, the app can be classified as a proximity tracing app, with end users voluntarily installing the app on their smartphones and voluntarily pushing their data through the app. The source code for various app components, the project website, and the feature development wish list are hosted in different folders on a public repository on GitHub. The source code is available to the public to view, and public interaction with the repository owners (developers) of the source code is possible with instructions provided on the repository in the form of a ReadMe file in each folder. Although the information in each folder’s ReadMe file is provided in English, support for requests written in German is also entertained. Public users are able to create their own version of the software or contribute to the existing software source code by following the instructions provided in each folder’s ReadMe file. Overall, the software in the repository is published under the open-source license Apache License version 2.0.

SORMAS is a public health surveillance and outbreak management tool used to capture data on cases and contacts of multiple infectious diseases [9]. It is typically installed and used by local health authorities and can therefore be classified as an outbreak management tool. The source code for the application is hosted on a public repository on GitHub and contains information about the repository in English. The source code is available to the public to view, and public interaction with the repository owners (developers) of the source code is possible, with instructions provided on the repository in the form of a ReadMe file. Public users are able to create their own version of the software or contribute to the existing software source code by following the instructions provided in the same ReadMe file. The software is published under the open-source license GNU GPL v.3. The results of this discussion are summarized and presented in Table 2.

We now proceed to discuss these results in more detail.

**Table 2:** Digital contact tracing solutions deployed in Japan and Germany in response to the COVID-19 pandemic.

Characteristic	COCOA^a^ (Japan)	HER-SYS^b^ and MyHER-SYS (Japan)	Corona-Warn-App (Germany)	SORMAS^c^ (Germany)
Classification	Proximity tracing	Outbreak management and symptom tracking	Proximity tracing	Outbreak management
Primary end users	Public	Health authorities and public	Public	Health Authorities
1. Source code published on a publicly accessible repository?	Yes (GitHub; English, Japanese) [27]	No	Yes (GitHub; English, German) [28]	Yes (GitHub; English) [29]
2. Source code accessible for public input, feedback, or interaction?	Yes (Process described in project ReadMe file hosted on GitHub) [30]	No	Yes (Process described in project ReadMe files [multiple] in separate folders hosted on GitHub) [28]	Yes (Process described in project ReadMe file hosted on GitHub) [31]
3. High level of independent or public source code customization possible as dictated by appropriate OSS license?	Yes (Process described in developer ReadMe file hosted on GitHub) [30]	No	Yes (Process described in developer ReadMe files [multiple] in separate folders hosted on GitHub) [28]	Yes | Process described in project ReadMe file hosted on GitHub [31]
License	Free to customize (Mozilla Public License version 2.0)	—^d^	Free to customize (Apache License version 2.0)	Free to customize (GNU GPL v.3)

^a^COCOA: COVID-19 Contact-Confirming Application.

^b^HER-SYS: Health Center Real-time Information-sharing System on COVID-19.

^c^SORMAS: Surveillance Outbreak Response Management and Analysis System.

^d^Not available.

## Discussion

### Principal Results

From Table 2, it is possible to see that proximity tracing tools for use by the public and outbreak management tools for use by health authorities have been implemented in both Japan and Germany. Japan additionally chose to integrate a symptom tracking tool (MyHER-SYS) for use by the public into their outbreak management tool (HER-SYS). In the case of Germany, the outbreak management tool SORMAS does provide software endpoints for the integration of a symptom tracking tool, although the actual application that does the symptom tracking does not form part of the core system and should be provided by an external stakeholder.

With this information, we may now answer our research question 1 (Which types of digital contact tracing solutions were developed and deployed by the Japanese and German governments in response to the COVID-19 pandemic?) by stating that a proximity tracing tool (COCOA) and an outbreak management tool (HER-SYS) with an integrated symptom tracking tool (MyHER-SYS) were developed in Japan (HER-SYS and MyHER-SYS are considered as 1 integrated system or solution, comprising the 2 functionalities going forward). Furthermore, a proximity tracing tool (Corona-Warn-App) and an outbreak management tool (SORMAS) were developed in Germany.

When further analyzing the responses to question 1 in Table 2, “Is the source code published on a publicly accessible repository?” we notice that 3 out of 4 (75%) solutions have published their source code on a publicly accessible repository, GitHub, with background information on the project provided at least in English.

Responses to question 2, “Is the source code accessible for public input, feedback, or interaction?” further indicate that 3 out of 4 (75%) solutions provide instructions to public users in the form of a ReadMe file. This file describes how the public may interact with the project on GitHub and provides an overview of the process they should follow to submit a request for a change or a new feature to the software. Similarly, question 3, “Is a high level of independent or public source code customization possible as dictated by an appropriate OSS license?” has 3 out of 4 (75%) solutions providing instructions to the public through a Project ReadMe file on how to interact with the project source code on GitHub, also providing an appropriate open-source license by which this is governed.

From this analysis, COCOA, Corona-Warn-App, and SORMAS can be classified as OSS, and HER-SYS (including MyHER-SYS) can be classified as CSS. When looking specifically at the 3 OSS solutions (COCOA, Corona-Warn-App, and SORMAS) and extracting their similarities, we notice that (1) open-source code, published on GitHub, provides project information at least in English. Furthermore, (2) the provision of a ReadMe file explaining how end users should interact with the software when requesting a new feature, as well as (3) a public developer ReadMe file explaining how public developers should interact with the software when developing new features, are provided. Lastly, (4) an appropriate open-source license has been chosen based on the individual needs of each solution. Table 3 summarizes the results.

With this information, we may now answer our research question 2 (How many of the digital solutions developed and deployed by the Japanese and German governments in response to the COVID-19 pandemic are open source?) by stating that 3 out of 4 solutions (75%) are open source.

Furthermore, in more generic terms, open-source code platform hosting choice (eg, GitHub), platform repository structure (eg, publicly accessible ReadMe files and licenses), and community or ecosystem engagement guidance and rules (eg, language support for interaction and ReadMe files) have been key elements of consideration in the public health OSS solution development strategies in response to the COVID-19 pandemic in both Japan and Germany.

Although the software source code and development process may be more transparent with OSS solutions as compared to the CSS solutions, the hosting, deployment, and operation of all live software systems in general (OSS and CSS) should also be taken into consideration. This is due to the fact that live user data will be stored, processed, and accessed in productive systems, with violations (in certain jurisdictions) being grounds for immediate termination of operation in addition to monetary or other penalties [32].

While the results in this paper may inform preliminary recommendations for future OSS initiatives, pandemic technologies, and strategies in public health, it is important to note that the database where the information processed by the software itself is stored or hosted in a live or production environment, is equally, if not more, important to consider.

**Table 3:** Open-source software (OSS) contact tracing solutions deployed in Japan and Germany: source code publication and management approaches similarity extraction.

Identifying question	COCOA^a^ (proximity tracing)	Corona-Warn-App (proximity tracing)	SORMAS^b^ (outbreak management)	Similarity
1. Source code published on a publicly accessible repository?	Yes (GitHub; English, Japanese) [27]	Yes (GitHub; English, German) [28]	Yes (GitHub; English) [29]	Yes (GitHub; English)
2. Source code accessible for public input, feedback, or interaction?	Yes (Process described in project ReadMe file hosted on GitHub) [30]	Yes (Process described in project ReadMe files [multiple] in separate folders hosted on GitHub) [28]	Yes (Process described in project ReadMe file hosted on GitHub) [31]	Yes (Project Read Me to guide users on the process to follow)
3. High level of independent or public source code customization possible as dictated by appropriate OSS license?	Yes (Process described in developer ReadMe file hosted on GitHub) [30]	Yes (Process described in developer ReadMe files [multiple] in separate folders hosted on GitHub) [28]	Yes (Process described in project ReadMe file hosted on GitHub) [31]	Yes (Project ReadMe to guide developers on the process to follow)
License	Free to customize (Mozilla Public License Version 2.0)	Free to customize (Apache License 2.0)	Free to customize (GNU GPL v.3)	Free to customize (choose license)

^a^COCOA: COVID-19 Contact-Confirming Application.

^b^SORMAS: Surveillance Outbreak Response Management and Analysis System.

### Limitations

This study is descriptive in nature, which comes with limitations. This extends itself to the application analysis (ie, a descriptive, case-based, classification approach which excludes specialist technical analysis), as well as the context in which these applications are analyzed (ie, a country-based comparative analysis which excludes broader policy, political, social, and economic factors, to mention only a few). The geographic limitation imposed on this research by selecting and analyzing solutions for contact tracing deployed in only Japan and Germany may further provide a limited view. This research nonetheless provides initial results that may serve as a point of reference for future studies covering a broader geographic, economic, or other area.

### Comparisons With Prior Work

To our knowledge, this research is novel in its approach and perspective of analyzing digital contact tracing solutions across not only different countries (Japan and Germany) but also different continents (Asia and Europe), between different types of contact tracing solutions (proximity tracing, outbreak management, and symptom tracking), in response to a global crisis (the COVID-19 pandemic). This contributes to the existing body of literature by setting a base for a culturally sensitive and transnational discourse on the role of digital technologies in crises.

### Conclusions

In response to the COVID-19 pandemic, various types of digital contact tracing solutions were developed and deployed by the Japanese and German governments in an attempt to interrupt COVID-19 transmission chains. In Japan, a proximity tracing tool (COCOA) and an outbreak management tool (HER-SYS) with an integrated symptom tracking tool (MyHER-SYS) were developed. In Germany, a proximity tracing tool (Corona-Warn-App) and an outbreak management tool (SORMAS) were developed, with the latter providing support for the integration of an optional external symptom tracking tool.

From these identified solutions, COCOA, Corona-Warn-App, and SORMAS were published as open source, indicating strong support by both the Japanese and German governments for OSS pandemic technology development in the context of public health. Open-source platform choice, platform repository structure, and community or ecosystem engagement are identified as key elements of consideration that may serve as preliminary guidance for other OSS pandemic technology development efforts.

Even with end users potentially knowing or finding out how their data are processed by OSS solutions (by having a look at the OSS source code hosted on public repositories), they do not necessarily know where the data that are being processed by the software are stored, or by whom the data are accessed or hosted. Despite the open nature of OSS solution source code, OSS solutions are therefore only as transparent as the live or production environment where their processed data are hosted or stored. Software development and live software hosting are thus 2 sides of the same coin and should be considered (at least) equally important in future pandemic technology development efforts. It is none the less arguable that OSS pandemic technology solutions for public health are a step in the right direction for enhanced transparency in the interest of the greater public good.

## Abbreviations

COCOA: COVID-19 Contact-Confirming Application
CSS: closed-source software
HER-SYS: Health Center Real-time Information-sharing System on COVID-19
OSS: open-source software
SORMAS: Surveillance Outbreak Response Management and Analysis System
